# Correction: Automatic emphysema detection using weakly labeled HRCT lung images

**DOI:** 10.1371/journal.pone.0220873

**Published:** 2019-08-01

**Authors:** Isabel Pino Peña, Veronika Cheplygina, Sofia Paschaloudi, Morten Vuust, Jesper Carl, Ulla Møller Weinreich, Lasse Riis Østergaard, Marleen de Bruijne

The last six authors, Sofia Paschaloudi, Morten Vuust, Jesper Carl, Ulla Møller Weinreich, Lasse Riis Østergaard, and Marleen de Bruijne, should not have been attributed equal contribution to this work.
